# Retraction of biomedical publications with Tunisian affiliation: causes, characteristics, and legislation regarding breaches of scientific integrity

**DOI:** 10.11604/pamj.2024.48.182.44793

**Published:** 2024-08-16

**Authors:** Amira Maoui

**Affiliations:** 1 University of Tunis El Manar, Faculty of Medicine of Tunis, Tunis, Tunisia

**Keywords:** Ethical issue, scientific fraud, research misconduct, Tunisia, plagiarism

## Abstract

**Introduction:**

breaches of research integrity have risen during these years. Tunisia´s stance regarding scientific integrity remains unknown. The aim of our study was to identify the reasons for the retraction of Tunisia-affiliated publications in the biomedical field, to describe the characteristics of these retractions, and to assess the position of Tunisian legislation regarding breaches of research integrity.

**Methods:**

I compiled up to November 3^rd^, 2023, and retracted biomedical papers using the PubMed and Retraction Watch databases. For each eligible retracted article, a descriptive study of the collected variables was carried out. These included the reasons for retraction, characteristics related to the article, authors, and journal.

**Results:**

the search identified 22 eligible publications. Reasons for retraction were categorized into six groups. Plagiarism accounted for 45.5% of cases. The first retraction dated back to 2005, with an average number of retracted publications being 1.22 and a median retraction time of 347 days. Among the retracted publications, 77.3% included a retraction notice. A post-retraction citation was found in 81.3% of cases. None of the retracted articles were written by a single author. An international collaboration was found in 27.3% of cases. Ninety-five point five percent of journals offered open access with 81.8% using a gold open access model. In terms of bibliometrics, eleven articles were published in highly reputed journals.

**Conclusion:**

Tunisia is not spared from breaches of scientific integrity. The controversies relating to the categories of breaches call for standardization. The legislative framework for this phenomenon also remains to be developed in Tunisia.

## Introduction

The ultimate goal of clinical research is to achieve excellence. Scientific excellence is based on three pillars: “ethics, deontology, and integrity” in order to assure a “responsible research” [1]. Scientific integrity remains nevertheless a challenging concept to define. It has recently been introduced in the French decree No. 2021-1572 of December 3^rd^, 2021 [2], “as the set of rules and values that must govern research activities to ensure their honest and scientifically rigorous nature”. Scientific integrity requires uncompromising intellectual integrity from all teams involved in research. It involves adherence to standards, laws, and regulations regarding research activities. It also requires a methodical management of data, experiments, and funds allocated to research. Finally, it claims respect for individuals engaged in research. If there is a particular interest nowadays that lends itself to the issue of scientific integrity, it is that misconducts are increasingly denounced during the retraction of publications. The causes are diverse: fraud, errors in good faith, and gray areas.

Publications related to retractions reported in some countries and the reminder of codes of conduct have multiplied in recent years. However, the situation in Tunisia regarding the concept of scientific integrity in the biomedical field remains unknown. Therefore, the objective of my study was to identify retracted publications with Tunisian affiliation, and to determine shortcomings in scientific integrity by noting the reasons for the retraction of each publication. To describe the characteristics of this retraction by studying those related to the corresponding author and the journal and finally to assess the position of Tunisian legislation regarding shortcomings in scientific integrity.

## Methods

**Study type:** a retrospective and descriptive study was carried out, focusing on the identification of retracted biomedical publications where at least one of the authors was affiliated with a Tunisian institution.

**Inclusion criteria:** the main inclusion criteria were the reason for the retraction of the biomedical publication with Tunisian affiliation and the description of the characteristics of retracted publications. The search was conducted on November 3^rd^, 2023, using the PubMed and Retraction Watch (RW) databases with no time filter introduced in the search query.

**Non-inclusion criteria:** retracted articles related to SARS-CoV-2 or COVID-19 were not included. This decision was made because COVID-19 publications are highly specific and may reflect unique dynamics related to the pandemic. By focusing on non-COVID-19 publications, the study provides a more representative overview of general retraction practices in Tunisia, applicable to a broader and non-pandemic research context.

**Exclusion criteria:** articles lacking clear reasons for retraction were excluded from the study. Retractions resulting from journal errors were ruled out to stay within the scope of scientific integrity. Additionally, duplicate publications were removed. Publications without a direct medical impact were also excluded.

### Data collection

**Strategy for retrieving retracted publications:** the PubMed query included keywords such as “retracted publication” or “retraction of publication”, affiliation “Tunisia”, excluding terms “COVID-19” or “SARS-CoV-2”. In Retraction Watch, the following sections were selected: Subject(s), Country(s), Nature of Notice, choosing respectively: (HSC) Health of Science OR (BLS) Basic Life Sciences, Tunisia, Retraction Notice.

### Analysis grid

**Study of parameters related to the publication and its retraction:** for each publication, the study included variables related to the paper, the corresponding author, the coauthors, and the journal. Parameters related to the paper included the reason for retraction, article type, time between publication and retraction date, presence of PubPeer comments, subject, biomedical specialty, study type (case series, meta-analysis, case report), nature of the sample when relevant, existence of a single author, and number of co-authors.

The categorization of the different reasons for retraction was aligned with Retraction Watch's classification. Total article citations, and pre- and post-retraction citations were determined using the Scopus database. Regarding the retraction itself, the mode (complete withdrawal, addition of a watermark, or a published notice) was studied. The initiator of the retraction was identified. Finally, measures taken by the journal towards the authors were examined.

**Study of parameters related to the authors and their affiliation:** parameters related to the author and affiliation were studied, focusing on the corresponding author. If this data was missing, the first author was considered as the corresponding one. The author's gender, affiliation (country and institution), total number of publications and citations, and h-index were collected. The corresponding author's scientific profile was determined by consulting Scopus. The affiliation of co-authors to countries other than Tunisia as well as their affiliation with academic, health, or research institutions were determined.

**Study of parameters related to the journal:** the study included variables such as the publishing group and two impact factors: the Impact Factor (IF) from the Journal Citation Reports (JCR) for the year 2022 retrieved from Web of Science by Clarivate Analytics (WoS), and the SCImago Journal Ranking (SJR) for the year 2022. The type of journal access, whether by subscription or open access (OA), and its different categories (green, gold, diamond) were searched on the Directory of Open Access Journals (DOAJ) website or directly on the journal's website. The membership status in the Committee of Publication Ethics (COPE) was searched on the COPE website. For non-member journals, a potential statement of adherence to COPE recommendations was featured directly on the journal's website. Compliance with the recommendations of the International Committee of Medical Journal Editors (ICMJE) was verified on the ICMJE website, or alternatively on the journal´s website.

**Statistical analysis:** a descriptive study of the collected data was conducted using the Excel software. Qualitative variables were described in terms of frequency and percentage, and quantitative variables in terms of frequency, median, minimum, and maximum.

**Tunisian legislation and research integrity:** in order to explore the stance of Tunisian legislation on breaches of scientific integrity, a dedicated paragraph was incorporated into the discussion section of this study. The review specifically focused on key Tunisian legislative documents that might govern biomedical publications with regards to maintaining scientific integrity.

## Results

**Selection of eligible articles and distribution by reason for retraction:** as of November 3^rd^, 2023, 22 eligible articles were identified (Figure 1). Among the retracted publications, 31.8% (7/22) had multiple reasons. Plagiarism (45.5%, 10/22) and duplication (37.3%, 6/22) were the most frequent reasons for retraction followed by errors in data, methodology, or results (22.7%, 5/22). Article plagiarism accounted for 60% (6/10), with 30% (3/10) attributed to text plagiarism and 10% (1/10) to image plagiarism. Four publications (18.2%) were retracted due to issues concerning authorship or scientific signature. Manipulation of images or data was detected in two instances (9.1%). Finally, only one publication (4.5%) was retracted due to a lack of Institutional Review Board approval.

**Figure 1:**
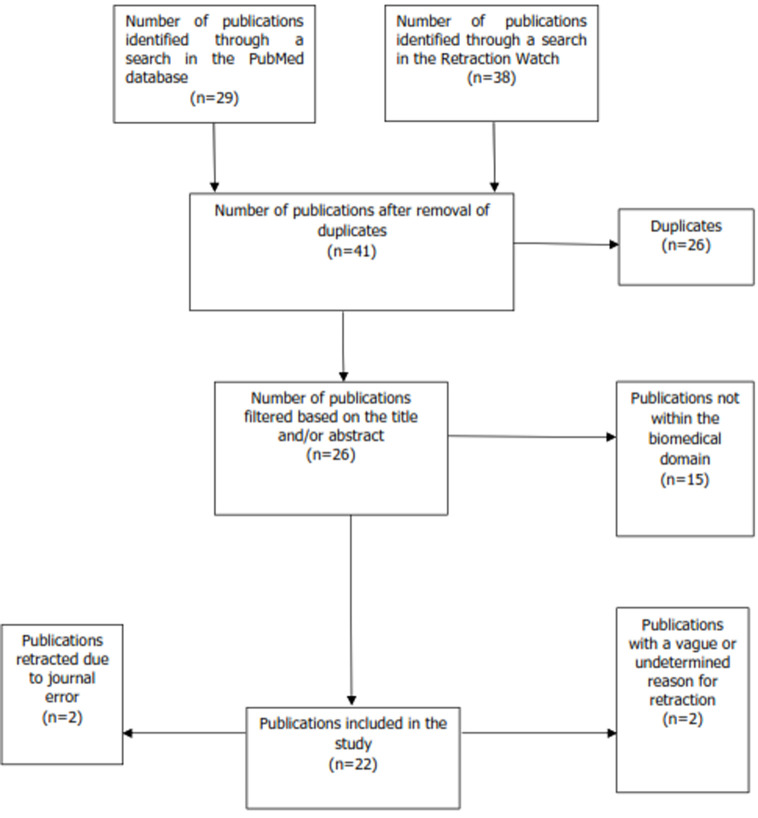
flowchart of retracted publications with Tunisian affiliation

**Frequency and retraction time:** the compilation of retracted publications spanned over 18 years and 10 months, resulting in an average of 1.22 retracted publications per year. The first identified retracted publication in PubMed and RW databases dated back to the year 2005. The time interval between publication and retraction ranged from 2 to 2466 days, with a median of 347 days.

**General characteristics related to the retracted publication:** approximately two-thirds of the retracted publications were research articles. The thematic focus of the publications was predominantly on biology (50%) (Table 1). Four articles received comments on PubPeer. One comment came from a “whistleblower”.

**Table 1:** qualitative characteristics of retracted publications

	Retracted publications	
	Number (n)	Percentage (%)
**Single author**		
Yes	0	0.0
No	22	100.0
**Type of the publication**		
Research article	16	72.7
Review article	1	4.5
Letter to the editor	2	9.1
Conference/abstract/paper	3	13.6
**Publication topic**		
Biology	11	50.0
Diagnostic	1	4.5
Clinical epidemiology	5	22.7
Clinical presentation	1	4.5
Therapeutic	3	13.6
Technology	1	4.5
**Specialty (discipline)**		
Oncology	4	18.2
Basic sciences	6	27.3
Gastroenterology	1	4.5
Urology	2	9.1
Neurosurgery	1	4.5
Hematology	2	9.1
Microbiology/infectious diseases	2	9.1
Dermatology	2	9.1
Radiology	1	4.5
Orthopedics	1	4.5
**Study type**		
Case series or retrospective cohort	17	77.3
Literature review or meta-analysis	2	9.1
Case report	1	4.5
Opinion or commentary on an article	2	9.1
**Nature of the sample**		
Human	12	54.5
Animal	5	22.7
Articles	2	9.1
Database (AI*)	1	4.5
**PubPeer comments**		
Yes	4	18.2
No	18	81.8
**Post retraction citation**		
Yes	13	59.1
No	3	13.6

AI*: artificial intelligence

Seventy-seven point three percent (17/22) of the articles included a published retraction notice available in PDF format, 83.3% (15/22) had a watermark, and 31.8% (7/22) were withdrawn from the journal. A conference published by an independent publisher included the mention “violation of publication principles” instead of the “retraction notice” mention. The term “erratum” was used as a title for retraction in two cases. Two journals have stated sanctions with a ban on the authors for future publications in the said journal (Table 2).

**Table 2:** characteristics related to the retraction notice

	Retracted publications	
	Number (n)	Percentage (%)
**Error admitted by the authors**		
No	13	59.1
Yes	2	9.1
Partially	2	9.1
Not mentioned	5	22.7
**Retraction initiated by the authors**		
No	13	59.1
Yes	4	18.2
Partially	1	4.5
Not mentioned	4	18.2
**Retraction initiated by the editor**		4.5
No	3	13.6
Yes	17	77.3
Partially	1	4.5
**No response from the authors**		13.6
No	5	22.7
Yes	1	4.5
Partially	2	9.1
Not mentioned	11	50
**Approval of retraction by the authors**		13.6
No	1	4.5
Yes	3	13.6
No response	1	4.5
Not mentioned	14	63.6
**Sanction for scientific misconduct**		
Not mentioned	19	86.4
Banning of the authors from the journal	3	13.6

**Characteristics related to the author and their affiliation:** the median number of authors was six [3-21]. Among the retracted papers, 81.3% were cited after their retraction, compared to 43.8% before the retraction.

The sex ratio was one (Table 3). Authors from multiple Tunisian institutions produced approximately 66.7% of the publications (14/22), and 27.3% (6/22) resulted from international collaboration. The medical school was associated with hospital structures only when it involved a research unit. This situation was found in two cases for Habib Bourguiba Hospital in Sfax. Academic affiliation was found in 63.6% of cases, corresponding to Tunis El Manar University, Carthage University, Sfax University, and Gabes University in 9.5%, 14.3%, 14.3%, and 4.8% of cases, respectively.

**Table 3:** qualitative characteristics related to authors of retracted publications

	Retracted publications	
	Number (n)	Percentage (%)
**Gender of the corresponding author**		
Male	11	50.0
Female	11	50.0
Country affiliation of the corresponding author* (binary proposition yes/no)		
Tunisia	21	95.5
Other than Tunisia	3	13.6
City of the corresponding author* (binary proposition yes/no)		
Tunis	11	50.0
Sfax	7	31.8
Sousse	2	9.1
Gabes	1	4.5
Bizerte	1	4.5
Gafsa	1	4.5
Presence of another country affiliation than Tunisia* (binary proposition yes/no)		
None	16	72.7
France	3	13.6
Italy	2	9.1
Qatar	2	9.1
Turkey	1	4.5
Switzerland	1	4.5
Affiliation structure for the author and co-authors* (binary proposition yes/no)		
Academic institution	14	63.6
Healthcare or hospital facility	13	59.1
Research laboratory	9	40.9

* : the sum of percentages exceeded 100% because a retracted article could be classified in multiple categories

The scientific profile of the corresponding author varied. It ranged from a single scientific publication with an h-index of zero to 443 publications and an h-index of 70. The median number of publications of the corresponding author was 15.5 (1-443). The number of citations as well as the number of documents citing the corresponding author varied from 115.5 to 18341 and 110 to 12353 respectively.

**Characteristics related to the journal:** fifty-nine percent of the journals that featured the retracted papers were among the top four prestigious journals [3]. The retracted papers were published in 22 different journals, and none of them were in a Tunisian journal. In almost half of the cases, the journals from which retractions occurred were categorized as Q1 and Q2 (Table 4). In terms of open science, only one journal provided diamond access. All journals claimed to adhere to the COPE ethical guidelines, with the majority also following the author guidelines established by the ICMJE. Only the IEEE Xplore journal appeared not to specify the adherence to ICMJE recommendations on its website.

**Table 4:** qualitative characteristics of journals in which retracted publications were published

	Retracted publications	
	Number (n)	Percentage (%)
**Publishing group**		
Elsevier	6	27.3
Springer Nature	3	13.6
Wolters Kluwer	2	9.1
Wiley-Backwell	3	13.6
Informa	1	4.5
Oxford University Press	1	4.5
Independent editor	5	22.8
Other: academic, institutional...	1	4.5
**Impact factor quartile**		13.6
Q1	7	31.8
Q2	4	18.3
Q3	7	31.8
Q4	1	4.5
**Access type (binary proposition yes/no**) *		
The journal requires a paid subscription	14	63.6
The journal offers an open access	21	95.5
Fees are required for authors or their institution (gold open access)	18	81.8
The journal offers self-archiving (green open access)	12	54.5
**COPE status**		
Member	16	72.7
Follow-up of recommendations	6	27.3
Neither member nor follow	0	0.0
**ICMJE status**		
Follow up of recommendations	21	95.5
No follow up	1	4.5

* : the sum of percentages exceeded 100% because a retracted article could be classified in multiple categories; COPE: Committee of Publication Ethics; ICMJE: International Committee of Medical Journal Editors

The bibliometric analysis of the journals in which the retracted articles were published was variable. The median Journal Citation Reports (JCR) impact factor in 2022 was equal to 3.4 (1.2-20.3) compared to the SJR impact factor, which reached 0.86 in the same year (0.32-4.93).

## Discussion

**Retraction reasons:** plagiarism was predominant in this study and found in 45.5% of cases. This percentage was comparable to that found in a study on retracted articles affiliated with Africa [4]. This situation is not specific to Tunisia, as the predominance of plagiarism could also be noted in studies carried out [5,6]. Article duplication was the second cause of retraction in the study (37.3%). Some authors define duplication as self-plagiarism and include it in the plagiarism category [7]. Others, however, separate duplication as a distinct cause [8]. The fifth identified reason for retraction was data and/or image manipulation, found in 9.1% of retracted articles affiliated with Tunisia. The rate of fraudulent conduct through data fabrication and falsification varies in the scientific literature. In the study conducted by Marco-Cuenca G *et al*. [9], scientific fraud through data falsification/fabrication ranged between 0% and 28.28% among 27 European Union countries. Several factors could explain research misconduct [9], with the main ones being the pressure of “publish or perish” [10], personality traits [11], factors related to competition for research funding [12], lack of experience or inadequate supervision [13], the absence of standardized policies governing conduct from countries, institutions, and publishers [14], and finally, socio-cultural factors [15].

**Frequency and retraction time:** this research found an average of 1.22 retracted articles per year, which is lower than the number reported among Indian authors [6]. The same study also reported that the first retracted article detected in the Scopus database dates back to 1996. In contrast, in my study, the first retraction revealed by PubMed was later, dating back to 2005.

The median retraction time in my research was 347 days. This time was almost double (591 days) among articles affiliated with Iran [8]. The retraction time varies depending on whether it involves fraud or error. According to Nath *et al*. [16], the average time for retraction in case of error was two years compared to three years in case of fraud. Explanations in the scientific literature attribute this difference to the fact that fraud allegations take longer to be established, primarily due to the associated investigative process [17].

**General characteristics of retracted articles:** the sex ratio of the corresponding author in this study was equal to one. Pinho-Gomes *et al*. [18] focused on the representation of female authors among retracted publications in biomedical sciences. Females represented 27% of first-listed authors. This study aligned with findings from other works suggesting that males might be more involved in fraud and research misconduct [19]. However, Fanelli's *et al*. [20] could not establish a clear relationship between gender and the predisposition to scientific misconduct.

Regarding the type of publication, research articles were most represented in the study (72.8%). Kamali *et al*. [21] reported similar percentages. Seventy-seven point three percent of the studies were case series or retrospective cohorts. The themes of retracted publications affiliated with Tunisia were mainly related to biology. Al-Ghareeb *et al*. [22] also noted a predominance of observational studies and basic sciences compared to clinical studies.

**Retraction mode, retraction notice, and compliance with COPE recommendations:** COPE conditions were not always met. Indeed, a retraction notice was found in 77.3% (17/22) of the articles in my study, compared to 98% (289/294) in McHugh *et al*. study [23]. In my study, retraction of articles originated exclusively from the authors in 18.2% of cases. This percentage has reached 63% in Wager *et al*. study [24]. Retraction can sometimes be initiated by what is called “whistleblowers”, who are usually readers alerting to misconduct. I identified one case on PubPeer. In Sharma *et al*. study [25], readers flagged 33 retracted papers out of 619.

**Retracted papers and open science:** scientific integrity is at the core of open science. Two aspects explain this relationship: data sharing and access to research results. Through data sharing, what matters for scientific integrity is the transparency of the data used and the reproducibility of the experiment. Access to results allows moving beyond the traditional peer-review system toward open peer review. This study revealed that 95.5% of journals provided open access. According to Chambers *et al*. [26], 26.1% of retracted articles were classified as open access. Lesk *et al*. study [27] preliminarily concluded that open access was less associated with fraud, especially when data were accessible. Open science undoubtedly offers a distinct advantage by providing access to information. However, there is a hidden downside to the coin found in the gold open-access model: article-processing charges, which are variable and explosive, have given rise to a new form of drift, that of predatory journals. Additionally, preprints pose a risk to the reader due to a lack of peer review, potentially leading to a loss of scientific quality.

**Retracted papers, authorship, and scientific signature:** authorship is connected to scientific integrity, as it raises questions about the definition of the term “scientific author”. In my study, issues related to authorship and scientific signature accounted for 18.2% of retractions. This retraction reason had reached a percentage of 19% in Dal-Ré *et al*. [28] study, which focused on retractions in the field of pharmacology.

In my study, the minimum number of authors was three. Collaboration could lead to conflicts and data falsification [29], diluting individual responsibility. Collaboration is challenging to define. It is not always correlated with the number of authors and may be underestimated in practices like ghost authorship or overestimated in honorary (gift) authorship. These two concepts, sometimes challenging to prove, were not identified in my study. The study conducted by Wislar *et al*. [30] on six high-impact medical journals identified 17.6% guest authors and 7.9% ghost authors.

**Retracted papers and affiliation:** Tunisian universities are included in the Shanghai ranking 2022-2023 [31]. Despite the prominent position of Tunis El Manar University in the field of health [32], none of the doctors from Tunis hospitals, whose publications were retracted, listed this university in their affiliation. This possibly indicates a lack of awareness about citation rules. Multiple national affiliations were observed in 66.7% of cases, and international collaboration was found in 27.3% of cases. Halevi *et al*. [33] identified various groups and trends related to multi-affiliation in academia. According to this author, the surge in global multiple affiliations is attributed to Tunisia's policies, aiming to promote scientific and technological collaborations with the European Union (EU) [33,34]. Indeed, the retracted papers showing international collaboration in my study were predominantly affiliated with EU countries: three cases with France, two with Italy, and one each with Switzerland and Turkey.

**Continued use of retracted papers:** the present study revealed that 81.3% of retracted publications were cited post-retraction, compared to 43.8% before retraction. This practice is not uncommon and has also been reported in Ghareeb *et al*. study [22]. When comparing literature data, we can observe that practices have not really evolved. Implementing applications like PubPeer in search engines could help counteract post-retraction citations. PubPeer detects comments on web pages, serving as a warning. The continued use of retracted articles has implications not only for the citing researcher but also for patients, especially in clinical or therapeutic studies. A cause for retraction deemed an unintentional mistake, is the utilization of incorrect data sourced from retracted articles. While my study did not uncover such instances, it is crucial to note that not every citation of a retracted article is necessarily erroneous if proper principles are adhered to [35]. Editors play, moreover, an essential role in ensuring that retraction notices align with COPE recommendations.

**Retracted papers and bibliometric indicators:** regarding productivity and scientific impact, the median h-index of corresponding authors in my sample was 5 (0-70). In Chambers *et al*. study [26], the h-index for the first author was 9 (0-80), and for seniors, it was 19 (0-78). Tunisian authors´ submissions in Q1 and Q2 journals accounted for 31.8% and 18.2% of cases, respectively. The study of Aspura *et al*. [36] found that retracted articles from Malaysian affiliation were published in journals classified Q1 in 43.4% and Q2 in 30.3% of cases. In my study, 27.3% of retracted publications were published in Elsevier journals, and 59% were among the “big four” publishers [3], represented by Elsevier, Springer Nature, Wiley, and Informa. The median Journal Citation Reports (JCR) Impact Factor (IF) from the Institute of Scientific Information (ISI) and the impact factor of the SCImago Journal Rank Indicator (SJR), with broader coverage than ISI, were 3.4 and 0.86, respectively, for the year 2022. Authors may choose such journals to enhance professional distinction and financial gain. Bibliometrics help assess a researcher's scientific activity, with institutions and governments also using them for evaluation purposes.

**Position of Tunisian legislation on scientific integrity breaches:** Tunisia's position on breaches of research integrity is not explicitly codified. While the concept of scientific integrity is not straightforwardly mentioned in Tunisian legislation, certain laws and decrees address related issues. One such decree is No. 2008-2422 of June 23^rd^, 2008, concerning plagiarism in higher education and scientific research [37]. This decree defines plagiarism and outlines sanctions for researchers who engage in it. In the event of plagiarism being substantiated, juries may decline to support student researchers, or refuse recruitment or promotion for the respective academic position. This decree, combined with quality initiatives by academic institutions, has led to the proliferation of codes of conduct and the implementation of anti-plagiarism software, such as PlagPrevent at the Faculty of Medicine of Tunis. However, subtle forms of plagiarism, like idea theft, are not addressed. The inadequacy of legal protection for scientific creation is a challenge faced in cases of plagiarism. Another relevant law is No. 2009-33 of June 23^rd^, 2009, amending and supplementing law No. 94-36 of February 24^th^, 1994, concerning literary and artistic property [38]. While mainly applicable to industrial domains, it establishes copyright and patent rights. The first article of this law lists the achievements covered by copyright. Two conditions are required to qualify as an achievement: formatting (the transition from the concept of an idea to its realization) and originality. Consequently, a scientific article can be considered an achievement (“written or printed works such as books, brochures, and other written or printed materials”). The resulting copyright includes both moral and patrimonial rights. In terms of moral rights, the researcher must uphold the right of disclosure, the right of authorship, and also preserve the material and intellectual integrity of the work. Concerning patrimonial rights, the researcher must obtain the author's permission before using their achievement. It is crucial to emphasize that, in terms of copyright and pursuant to article 14 of law No. 2000-68 dated July 17^th^, 2000, amending certain provisions of law No. 96-6 dated January 31^st^, 1996, related to scientific research and technological development, the provision states that any invention or discovery made by a researcher in the public sector is deemed to be the property of the state, represented by the overseeing institution.

Concerning research data, the reproducibility of experiments and results is an important concept in scientific integrity. It is associated with the concept of the laboratory notebook but also with access to research data and information. A research conducted with integrity should be transparent and reproducible. This leads us to discuss the management, dissemination, and storage of research data. In the era of big data and open science, it is important to regulate practices to prevent breaches of scientific integrity. Despite the existence of some general texts, notably government decree no. 2021-3 of January 6^th^, 2021, on open public data [39], framed by the standards set by the “Instance Nationale de Protection des Données Personnelles INPDP” in the 2004 law [40], the field of open science remains unregulated.

Regarding corruption prevention, the declaration of conflicts of interest plays a crucial role in scientific integrity. There is no explicit text regulating physician researchers or scientists in Tunisia. They can be however considered as public agents, falling under various legislations such as decree No. 2014-4030 of October 3^rd^, 2014, approving the code of conduct and ethics for public agents [41], and law no. 2018-46 of August 1^st^, 2018, concerning the declaration of assets and interests, the fight against illicit enrichment, and conflicts of interest [42]. Tunisia has established a Governance and Anti-Corruption Authority, and various laws require presidents of universities, deans, directors, and heads of laboratories to declare their assets and interests. Sanctions range from fines to imprisonment, but they need further clarification in the context of scientific and medical publications. Masmoudi *et al*. [43] underscores that the methods of regulating conflicts of interest in the Tunisian health sector, encompassed by flexible ethical laws on one hand and stringent legal measures on the other, remain either “difficult to define” or “relatively precise”, necessitating “the deployment of enormous means”. This specialist in public law at the Faculty of Legal, Political, and Social Sciences of Tunis, emphasizes the imperative of legal intelligence to effectively regulate conflicts of interest in the Tunisian health system.

**Strengths and weaknesses of the study:** this study is original in that no previous research has profiled Tunisian medical scientists in terms of scientific integrity. However, some methodological limitations should be acknowledged. The research was limited to the PubMed database, which considers only indexed articles. Expanding the search beyond PubMed and Retraction Watch would have provided a more comprehensive view of the phenomenon. Another limitation is associated with post-retraction citation. This can be either positive or negative, and thus, the resulting consequences may vary. Additionally, a third limitation was related to the determination of the COPE status of journals. It would have been interesting to consult the journal's policy to precisely determine which criteria were chosen from the recommendations of the COPE.

## Conclusion

Tunisia is not exempt from breaches of scientific integrity. Although the number of 22 cases in the biomedical field may seem small, it is important to contextualize this finding by considering the databases used in the research and understanding Tunisian publication practices. In terms of Tunisian legislation and scientific integrity, there is still a long way to go the question arises: are we demonizing science, or are we too lenient? Will there come a day when using artificial intelligence, we will need to create a robot to be our conscience, reminding us of fundamental principles and the morality of what is right and wrong? To conclude, I suggest always keeping in mind what Rabelais, a renaissance physician and humanist writer, said: “Science without conscience is only the ruin of the soul”.

### What is known about this topic

- Studies suggest that scientific misconduct is a significant issue in medical research, with various forms such as falsification, fabrication, and plagiarism occurring at notable rates and leading to serious consequences;
- Studies suggest that factors influencing scientific integrity breaches include a lack of research integrity policies, personal and institutional bias, external pressures, and organizational variables such as time and resources.

### What this study adds

- Patterns of retraction in Tunisia: the study identifies plagiarism and duplication as the leading causes of retraction among Tunisian-affiliated publications, offering a detailed analysis of the retraction landscape in the country;
- Impact and characteristics: it examines the temporal trends, author affiliations, and the common features of retracted publications, shedding light on the research dynamics and institutional vulnerabilities in Tunisia;
- Recommendations for scientific integrity: the findings underscore the need for stronger legislative frameworks and clearer authorship guidelines to reinforce scientific integrity and reduce retraction rates.
